# Extracellular vesicle signalling in atherosclerosis

**DOI:** 10.1016/j.cellsig.2020.109751

**Published:** 2020-11

**Authors:** E. Charla, J. Mercer, P. Maffia, S.A. Nicklin

**Affiliations:** a Institute of Cardiovascular & Medical Sciences, University of Glasgow, Glasgow, UK; b Institute of Infection, Immunity and Inflammation, University of Glasgow, Glasgow, UK; c Department of Pharmacy, University of Naples Federico II, Naples, Italy

**Keywords:** Extracellular vesicles (EVs), Atherosclerosis, Coronary artery disease (CAD), microRNA (miRNA), coronary artery disease, (CAD), cardiovascular disease, (CVD), endosomal sorting complex required for transport, (ESCRT), extracellular vesicle, (EV), intraluminal vesicles, (ILV), late sorting endosome, (LSE), low density lipoprotein, (LDL), smooth muscle cell, (SMC), endothelial cell, (EC), matrix metalloproteinase, (MMP), multi-vesicular body, (MVB)

## Abstract

Atherosclerosis is a major cardiovascular disease and in 2016, the World Health Organisation (WHO) estimated 17.5 million global deaths, corresponding to 31% of all global deaths, were driven by inflammation and deposition of lipids into the arterial wall. This leads to the development of plaques which narrow the vessel lumen, particularly in the coronary and carotid arteries. Atherosclerotic plaques can become unstable and rupture, leading to myocardial infarction or stroke. Extracellular vesicles (EVs) are a heterogeneous population of vesicles secreted from cells with a wide range of biological functions. EVs participate in cell-cell communication and signalling via transport of cargo including enzymes, DNA, RNA and microRNA in both physiological and patholophysiological settings. EVs are present in atherosclerotic plaques and have been implicated in cellular signalling processes in atherosclerosis development, including immune responses, inflammation, cell proliferation and migration, cell death and vascular remodeling during progression of the disease. In this review, we summarise the current knowledge regarding EV signalling in atherosclerosis progression and the potential of utilising EV signatures as biomarkers of disease.

## Highlights

• Extracellular vesicles are secreted under physiological and pathological conditions.
• They participate in cellular signalling via transfer of their cargo.
• They may contribute to all stages of atherosclerotic lesion progression.
• Circulating extracellular vesicles could serve as biomarkers.
• Extracellular vesicles could be used as therapeutic tools.

## 1 Pathophysiology of atherosclerosis

**Fig. 1:**
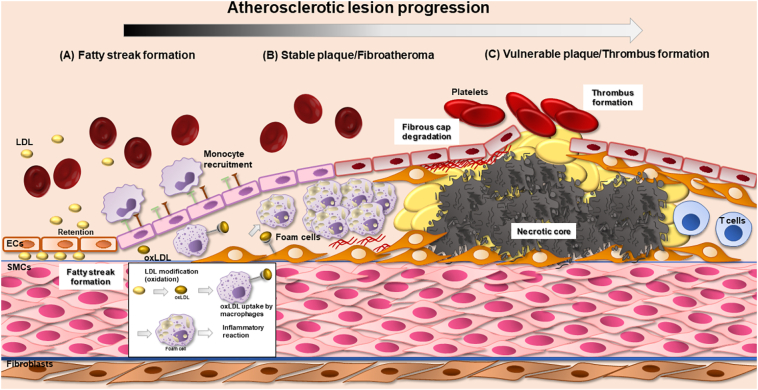
Atherosclerotic plaque formation. (A) Lipoprotein retention to the vascular wall and disturbed flow activate the expression of adhesion molecules (vascular cell adhesion molecule-1 (VCAM-1), intercellular adhesion molecule-1 (ICAM-1)) on endothelium with subsequent monocyte recruitment to the vessel wall. Monocytes differentiate to macrophages, take up oxidised low density lipoprotein (oxLDL), form foam cells and a pro-inflammatory reaction is activated. Vascular modifications result in SMC migration to the subendothelial space. (B) A stable plaque is formed of a lipid core and the accumulation of necrotic cells as foam cells undergo apoptosis and necrosis. SMC secrete macromolecules like collagen, elastin, fibronectin and extracellular matrix facilitating fibrous cap formation. (C) Thin fibrous cap results in a vulnerable plaque prone to rupture and secondary complications like thrombus formation. Macrophage proteolytic activity with matrix metalloproteinases (MMPs) being the main proteolytic enzymes has been associated with plaque rupture.

The term cardiovascular disease (CVD) is used to describe the pathologies affecting the heart or circulation, including heart failure, coronary artery disease (CAD), stroke, hypertension and atherosclerosis. Atherosclerosis is a chronic inflammatory disease characterised by lipid-laden plaques developing in the vessel wall and can cause myocardial infarction (MI), stroke, unstable angina and sudden cardiac death [[1], [2], [3]]. Despite significant advances in pharmacological treatments and surgical interventions over the past 20 years, atherosclerosis remains the leading cause of vascular death worldwide [4]. Atherosclerosis is no longer considered merely a lipid storage disease as studies have reported inflammatory mechanisms that participate in lesion progression such as leukocyte recruitment to the lesion site [5,6]. Leukocytes in the plaque can secrete growth factors inducing SMC proliferation in advanced lesions [7]. Low density lipoprotein (LDL) retention in the arterial wall is considered the initial step of the disease [8,9]. Ross at al, in 1973 formulated the Respone to Injury theory where atherosclerosis results from endothelial injury [10,11]. Williams and Tabas in 1995, first formulated the Response-to-Retention Hypothesis of Early Atherogenesis (suggesting that lipoprotein retention in the vessel wall was the initial step of the disease), a theory that originated from the Anichkov and Khalatov hypothesis of cholesterol retention in the vessel wall in atherosclerotic plaque formation [12,13]. The current knowledge regarding atherosclerotic lesion progression is summarised in Fig. 1. In the vessel wall, LDL undergoes several modifications such as oxidation, enzymatic cleavage or aggregation [9,14]. Studies have shown that oxidised low density lipoprotein (oxLDL) can act as an antigen and initiate an immunological response via the generation of antibodies against oxLDL [15]. Macrophages take up oxLDL to remove it from the arterial site but the result is the formation of foam cells and subsequent activation of an inflammatory response [14]. Clinical data have confirmed the pathological role of LDL levels in disease progression and the subsequent reduced cardiovascular risk after patients are prescribed lipid lowering therapy (the mechanism of action of lipid lowering therapies such as statins involves reduced cholesterol levels) [8,16,17]. Taking into consideration the inflammatory response present in all steps of the disease, LDL retention occurs along with endothelial cell (EC) activation and dysfunction triggered by cytokines resulting in the expression of adhesion molecules [5]. Lipid lowering therapies are successful, however, the burden of recurrent cardiovascular events exists [5]. Two recent clinical trials, the Canakinumab Anti-inflammatory Thrombosis Outcome Study (CANTOS) and the Colchicine Cardiovascular Outcomes Trial (COLCOT) have shown that targeting inflammation may be a viable approach to lower the ratio of recurrent cardiovascular events [18,19].

### 1.1 Fatty streak and lesion progression

High levels of LDL particles in plasma are linked with coronary events [20,21]. Atherogenic apoB-containing lipoprotein retention to atheroprone arterial sites is the initial step in fatty streak lesion formation (Fig. 1) [[22], [23], [24], [25]]. Atheroprone sites are often branch points of the arteries subjected to non-laminar and turbulent blood flow and display predisposition to lesion development [26]. Atheroprone flow activates NF-κB in ECs inducing expression of inflammatory cytokines and sets the scene for an atherogenic environment [27]. Upon inflammatory stimuli, ECs start to express vascular cell adhesion molecule-1 (VCAM-1) and intercellular adhesion molecule-1 (ICAM-1), facilitating monocyte migration into the intact vessel wall [[28], [29], [30]]. LDL is oxidised via enzymatic or non-enzymatic pathways to form oxLDL [9,31]. Subsequently, CD11b + monocytes upregulate scavenger lipoprotein receptors such as Scavenger Receptor Class A type I and II (SR-AI/II), Scavenger Receptor Class B, type I (SR-BI) and CD63, acquire a macrophage (Mac3+) phenotype and transmigrate into the sub-endothelial space and accumulate oxLDL [[32], [33], [34]]. This change of macrophage phenotype into foam cells, is the hallmark of fatty streak lesions, the earliest sign of atherosclerotic plaque development [32,33]. Furthermore, the inflammatory reaction is enhanced as monocytes or macrophages present antigenic epitopes of oxLDL to B-cells, inducing the formation of antibodies to oxLDL and an immune reaction towards deposited oxLDL [35].

The end result leads to several vascular modifications (Fig. 1). SMC also accumulate in the atheroma and secrete macromolecules such as fibronectin which contribute to the formation of the fibrous cap and increase plaque stability [36]. SMC retain phenotypic plasticity, enabling them to undergo a switch from a contractile, quiescent phenotype to a synthetic, proliferative and migratory phenotype [37]. This phenotype alteration to dedifferentiated SMCs is critical for the pathogenesis of atherosclerosis (intimal lesion progression) and other vascular diseases characterised by intimal thickening [36]. Interestingly, recent lineage tracing studies have shown that dedifferentiated SMC that lack normal SMC markers can express macrophage-like markers such as CD68, Mac2 and LGALS3 [36,38,39]. SMC also express scavenger receptors participating in foam cell formation in early atherogenesis and express proteoglycans that promote LDL retention [40].

### 1.2 Atheroma formation

Typical atheromas contain a lipid core, apoptotic macrophages forming a necrotic core and a developing thick fibrous cap facilitated by SMC production of collagen, elastin, fibronectin and extracellular matrix [37]. Macrophage activation leads to the release of several cytokines, their transformation into foam cells and their sequential necrosis [41]. Activated macrophages release further inflammatory stimuli and enhance the necrotic core formation in advanced atheromas [41].

### 1.3 Calcification

Vascular calcification is the process of accumulation of minerals to intima or media of the vessel wall, and is present in the late stages of atherosclerosis [42]. Plaque calcification is linked to plaque stability, stable fibrous cap formation and reduced macrophage infiltration [[43], [44], [45]]. Shaalan et al., suggested that quantification of calcified area could be a novel marker to assess cerebrovascular ischemic event risk [44]. However, older studies have linked the amount of calcium with cardiovascular burden and the severity of the disease [46]. Microcalcification in the fibrous cap can promote plaque instability and rupture, but calcified plaques are less prone to rupture highlighting a dual role for calcification in atherosclerosis [47]. A possible explanation comes from high-resolution imaging and clinical data, showing that microcalcification is found in areas with low levels of collagen (collagen is responsible for formation of the thick fibrous cap), whereas heavily calcified regions are bordered with collagen fibres [48]. SMC retain phenotypic plasticity but transdifferentiation to chondrocytic, osteoblastic and osteogenic phenotypes marks the beginning of the calcification process [[49], [50], [51], [52]]. Molecular mechanisms regarding the calcification process are still poorly understood.

### 1.4 Vulnerable plaques

Vulnerable and ruptured plaques are characterised by a thin fibrous cap and expanding necrotic core due to macrophage and SMC apoptosis [53]. Macrophage proteolytic activity has been implicated in plaque destabilization and degradation of the stable fibrous cap [[54], [55], [56]]. Detection of proteolytic enzyme activity such as matrix metalloproteinases (MMPs) (MMP-8, MMP-9 and MMP-12) is reported to predict plaque rupture and thrombus formation, a major manifestation of atherosclerosis implications [57,58]. MMPs are proteolytic enzymes and dedradation of extracellular matrix including collagen by MMPs has been extensively reported [57]. MMP-8 for example is a well known collagenase and MMP-9 a well known gelatinase and reports suggest they contribute to plaque destabilization and rupture by degradation of the fibrous cap [59,60]. The exact mechanism for how increased proteolytic activity affects plaque rupture is still poorly understood.

## 2 Extracellular vesicles

### 2.1 EV biogenesis

Extracellular vesicles (EVs) are small membrane-bound vesicles that are secreted from all cell types and were first thought to remove unwanted membrane proteins from cells [61]. In the 20th century, researchers started to gather evidence for the presence of EVs, and the term extracellular vesicles was first used in 1971 [[62], [63], [64]]. First, EVs were believed to remove unwanted proteins from cells but in recent years their actions have been found to mediate a variety of biological functions, thus, new insight into the role of EVs has been revealed. EVs can carry lipids such as cholesterol, sphingomyelin, phosphatidylserine, proteins, and genetic material (particularly RNA and small non coding RNAs) [65]. They can transfer their cargo to other cells and are implicated in many physiological processes including cell apoptosis, immune responses, inflammation, and coagulation [65]. Many studies have focused on unravelling their role in different diseases, including CVD and atherosclerosis [[66], [67], [68], [69]].

Classification of EVs is a relatively new process and they can be categorised based on their size and biogenesis [70]. Based on their biogenesis EVs can be further categorised as exosomes, microvesicles and apoptotic bodies [47]. Microvesicles represent large particles with size range 100 nm up to 1 μm [47]. In contrast, exosomes, with size range from 30 nm to 150 nm, are believed to be particles of endosomal origin through the endosomal sorting complex required for transport (ESCRT)-dependent pathway, as many endosomal proteins have been identified on their membranes [[71], [72], [73]]. Another EV subtype are apoptotic bodies with sizes up to 5 μm, released during apoptosis (apoptotic body formation reviewed in [74]). Two subtypes of EVs, exosomes and microvesicles, are being studied extensively regarding their role in signalling in CVD and whether they could be utilised as biomarkers to detect the early stages of the disease.

**Fig. 2:**
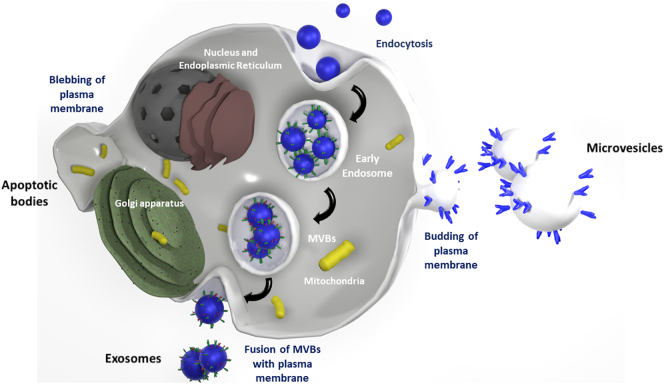
EV Biogenesis. EV classification is based upon the particle size and their biogenesis. Apoptotic bodies are large vesicles and are formed after blebbing of the plasma membrane. Microvesicles are a product of outward budding of the plasma membrane. Exosomes are smaller endosomal vesicles and released upon fusion of the MVBs with the plasma membrane. The figure was generated with Blender 2.8 which is released for free use under a GNU General Public License (GPL).

The biogenesis of microvesicles and exosomes has similarities, but the main difference is that microvesicles come from shedding of the plasma membrane (Fig. 2). Biogenesis of exosomes is initiated by invagination of the plasma membrane where active molecules are captured by endocytosis forming a structure called the early-sorting endosome (ESE) which then matures into late-sorting endosomes (LSEs) [75]. Next, proteins of the ESCRT pathway are recruited to endosomes to facilitate formation of multivesicular bodies (MVBs), through inward budding of the endosomal membrane. During this time, pre-cursors to exosomes termed intraluminal vesicles (ILVs) are enriched with cholesterol and other lipids such as sphingomyelins [[76], [77], [78], [79], [80]]. The ESCRT pathway then also transfers functional proteins including Tumor Susceptibility Gene 101 (TSG101) and Suppressor of K+ Transport Growth Defect 1 (SKD1) into ILVs and an ESCRT subunit is then responsible for ILVs maturing into exosomes [[81], [82], [83]]. MVBs are full of ILVs which then later mature into exosomes. MVBs can either fuse with lysosomes and be degraded or they can follow an alternative pathway and fuse with the plasma membrane and release their cargo, called exosomes, into the extracellular space [84]. Studies suggest that exosome secretion is linked with removal of parts of the plasma membrane during plasma membrane remodeling and this may be responsible for the presence of cell-membrane specific proteins on the exosome surface, contributing to maintaining cellular homeostasis and furthermore MVBs are associated with receptor downregulation [85,86]. Lysosome degradation of unwanted or damaged proteins is a way to maintain cell homeostasis [87]. In both pathways, MVB fusion with either the cell membrane or the lysosome, the anticipated effect is removal of proteins from the cell and is achieved with the use of similar pathways, molecular components and organelles [88]. A clear mechanism for how either MVB fusion with the lysosome or MVB fusion with plasma membrane for later exosome secretion interact is not clear. Key questions still remain unknown regarding whether the same population of MVBs participates in both processes.

Generation of microvesicles via outward plasma membrane shedding is also a physiological process. Generally, microvesicles are larger vesicles than exosomes, although there is an overlap in their size profiles. Microvesicle biogenesis begins upon nucleation, whereby tetraspanin proteins and lipids on the plasma membrane surface cluster [89]. The outward budding of the plasma membrane to release microvesicles requires changes in the architecture of the cytoskeleton and elevated levels of free intracellular Ca^2+^ act as a second messenger to ensure the release of microvesicles [89]. As the cytoskeleton is exposed, proteins and genetic material can internalise inside the lumen of the microvesicle [90]. Two pathways have been reported to participate in the budding and pinching off of the membrane for microvesicles to be generated, the ESCRT pathway, which is also involved in exosome generation, and a signalling cascade involving ARF6 through activation and recruitment of PLD/ERK and phosphorylation of MLCK [91,92]. The distinct differences in exosome and microvesicle generation account for their different surface protein repertoire and also their cargo. Generation of microvesicles through outward plasma membrane shedding is a physiological process occurring in all cell types [89].

### 2.2 EV heterogeneity

Discussion around the diverse biological functions of EVs is extensive with studies reporting contradicting results for the same EV population. It is crucial though to mention that EV heterogeneity makes EV research challenging. EVs can differ in their size, cargo, biological effect, cellular origin, and the microenvironment in which the cells were cultured, as well as in their isolation method. Many factors that effect EV generation and secretion, including budding of the cell membrane and pinching off of ILVs or microvesicles can lead to exosomes or microvesicles with subpopulations of distinct size range [93,94]. The microenvironment can also have an impact on the cargo of EVs and, for example, determine specific miRNAs to be encapsulated inside the EVs [95]. A study on quantitative and stoichiometric analysis of miRNA cargo revealed that exosomes contain low number of miRNA copies [96]. However, an increasing number of studies report functional EV-mediated transfer of miRNAs to recipient cells [[97], [98], [99]]. Furthermore, a proteomic analysis of isolated EVs demonstrated the existence of subpopulations within EVs with distinct protein markers [100]. These data indicate the existence of subpopulations of EVs with distinct cargo and functions and can explain their varied and contrasting effects on recipient cells.

### 2.3 EV isolation methods

EVs can be found and isolated from many biofluids, including serum, plasma, saliva, urine, amniotic fluid, breast milk, cerebrospinal fluid, nasal secretions, culture media as well as from cells in tissues [[101], [102], [103], [104], [105], [106], [107], [108], [109]]. Taking into account EV heterogeneity and the fact that isolated particles come from biological fluids contaminated with non-vesicular components such as cell debris, proteins and other non-EV particles, high purity EV yield with adequate separation of EV subpopulations is challenging [110]. A major limitation of many EV isolation protocols is the co-isolation of lipoproteins, protein aggregates or non-EV particles along with the EV population. Co-isolation of lipoproteins like LDL or high density lipoprotein (HDL) particles comprises a challenge when working with serum or plasma as abundant lipoprotein levels are found in blood. In a recent study using a density gradient method of isolation it was shown that EVs were co-isolated with HDL particles which have a similar density between 1.063 and 1.21 g/mL [111]. LDL particles are no different as studies show they also co-isolate with EVs [112]. Although, lipoprotein depletion with antibodies has been proposed, it was shown that a significant number of EVs are lost with that method [113]. The same limitation applies to non-EV membrane shedding particles such as matrix vesicles which are known to present EV characteristics [114].

To this day, there is no gold-standard method for EV isolation. Many studies have compared isolation techniques for their efficacy and yield purity [110,115]. Briefly, the main EV isolation methods include: differential ultracentrifugation (UC), density-gradient separation, polymer-based precipitation, immunoselection and size exclusion chromatography (SEC) [[116], [117], [118]]. It is worth mentioning that each of these methods comes with its own limitations [119]. One of the most common techniques used for EV isolation is UC, whereby particles with different sizes and densities will show different sedimentation characteristics [[120], [121], [122]]. Inconsistencies and low method reproducibility regarding EV isolation via UC have been reported and many factors can be responsible for this including centrifugation speed, rotor type, and temperature [[110], [111], [112], [113], [114], [115], [116], [117], [118], [119], [120], [121], [122]]. SEC allows the separation of particles based on their size, although the separation between non-EV particles and co-isolation of lipoproteins are limitations of this method [110,119]. Comparative studies between the two methods suggest that SEC allows EV yield with higher concentrations but lower purity compared to UC [110,119]. Polymer-based precipitation (PBP) methods exploit the use of a polymer precipitation agent to reduce EV solubility and allow EV isolation along with co-isolation of non-EV particles and proteins [119,123]. Although, this isolation method does not affect EV integrity it has been suggested that the precipitation agent may interfere with downstream analyses [123,124].

Due to the increasing interest in researching the functions of EVs, the need for standardization of an isolation method is fundamental. Many factors may influence the choice of isolation method such as the tools available to the researcher, downstream analysis required and starting material. For example, SEC attracted a lot of interest as studies have suggested that preservation of EV functionality is greater compared to the classical UC method [110,125]. As discussed previously, absolute separation of EV populations is challenging as EV subpopulations can overlap in size. Sluijter et al., in a Position Paper from the Working Group on Cellular Biology of the Heart of the European Society of Cardiology make recommendations for optimal isolation methods depending on the starting material [126]. A combination of two isolation methods has been proposed to overcome the low purity of one isolation method alone but at the expense of reduced EV yield [119,127]. Therefore, until a universal isolation method is achieved, great consideration regarding the method used to isolate EV populations should be given in order to assess the biological effects of isolated EVs.

## 3 EV signalling in atherosclerosis progression

**Fig. 3:**
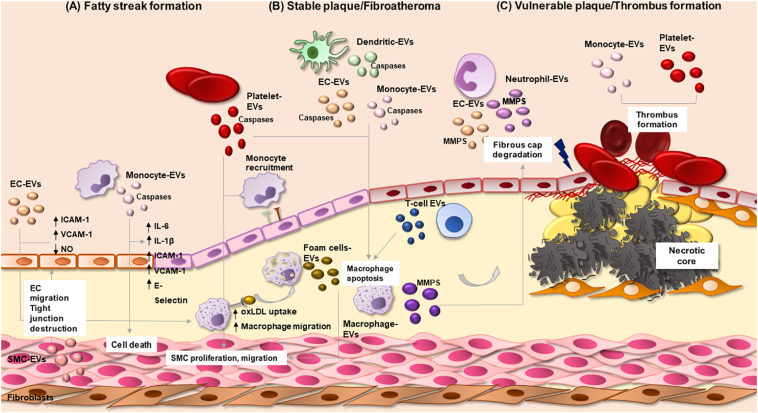
EV signalling in vascular inflammation and atherosclerosis. Brief schematic representation of EVs and their roles in the steps of atherosclerotic lesion progression. (A) During fatty streak formation, EC-derived EVs promote endothelial ICAM-1, VCAM-1 expression, reduction of NO production, oxLDL uptake by macrophages and macrophage migration. Monocyte-derived EVs induce vascular inflammation (expression of IL-6, IL-1β), upregulation of adhesion molecules in ECs (ICAM-1, VCAM-1 and *E*-selectin) and vascular cell death. Foam cell-EVs and platelet-EVs can induce SMC proliferation and migration, aggravating the progression of the disease. Crosstalk between cells is fundamental; SMC-EVs can promote EC migration and via miRNA transfer promote tight junction destruction. (B) Dead cells accumulate in the plaque's necrotic core. Platelet, endothelial, dendritic and monocytic-derived EVs encapsulate cell death related proteases: caspase-1 and caspase-3 that can induce macrophage apoptosis. Monocyte-EVs can promote SMC death via caspase-1 and participate in formation of the atherosclerotic plaque. (C) Weakening of the fibrous cap is the main cause of plaque rupture. EVs from various sources (macrophage, neutrophil, endothelial) encapsulate MMPs and may degrade extracellular matrix and destabilize the plaque. Platelet and monocyte derived EVs can also enhance thrombus formation.

EVs are reported to be present in both intimal lesions in developing plaques and in advanced plaques suggesting that they participate in the initial and final stages of plaque formation in humans [[128], [129], [130]] (Fig. 3). Characterisation of plaque thrombogenic EVs demonstrate that the majority originate from leukocytes (52%), followed by macrophages (29%), erythrocytes (27%), lymphocytes (15%), SMCs (13%) and ECs (8%) [129].

### 3.1 EVs and fatty streak and lesion progression

EC activation is among one of the first steps in intimal lesion formation. Levels of circulating EC-derived microvesicles are increased in patients with underling cardiovascular risk factors [131,132] including smoking [133,134], hyperlipidaemia [135] and high blood pressure [136,137]. Endothelial dysfunction or activation induces the production of EVs from ECs [138]. EC-derived EVs have gained interest as indicators of pathology but studies have also reported that they can bind platelets and monocytes and thus regulate thrombus formation [139,140]. EC-derived EVs seem to have ambiguous roles in the initial steps of the disease, being reported to signal in processes that promote endothelial dysfunction and the development of atherosclerosis as well as mediating protective signalling. Densmore et al., showed that EC-derived EVs supressed nitric oxide (NO) production in ECs and aggravated endothelial function [141,142]. Oxidised lipoproteins can also alter the EC-derived EV cargo favouring proinflammatory molecules which can induce the expression of adhesion molecules such as ICAM-1 and VCAM-1 [[143], [144], [145]]. Interestingly, Chatterjee et al., also showed that EC-EVs can affect the initial steps of the disease by aggravating endothelial barrier dysfunction [146]. EC-derived EVs induced destabilization of tight junction proteins via c-Src kinase transfer causing barrier function disruption [146]. Another study showed that miR-92a transfer from EC-derived EVs to macrophages resulted in increased LDL uptake and macrophage migration, while inhibition of miR-92a expression abolished these effects, indicating a regulatory role for miR-92a in vascular disease [99].

EC-derived EVs exert atheroprotective effects too. Interestingly, EC-derived EVs can regulate vascular homeostasis as miR-126 transfer to recipient ECs promotes EC migration and miR-222 transfer reduces ICAM-1 levels on ECs [147,148]. In both studies, reduced levels of miR-126 and miR-222 in EVs were observed in patients with CAD [147,148]. Interestingly, non-EV transfer of miR-126 has an atherogenic impact on SMCs by inducing proliferation and apoptosis of SMC, but these effects were abolished under atheroprotective laminal shear stress (LSS) [149]. Regulation of EC migration is vital for re-endothelialisation after vascular injury. Krüppel-like Factor 2 (KLF2) is a transcription factor implicated in EC behaviour [150,151]. EC-derived EVs from KLF2 expressing cells promoted an anti-inflammatory response and repressed monocyte activation by reducing the expression of the inflammatory miR-155 [144]. Another study identified that endothelial EV-mediated transfer of miR-10a repressed monocyte activation via NF-κB downregulation [152]. In vivo the same EV population reduced plaque size and reduced M1 macrophage phenotype with a shift to the M2 anti-inflammatory macrophage phenotype [144]. These findings show that inhibition of miR-155 could be a new target for atherosclerosis treatment. Furthermore, it was also reported that EC-derived EVs from KLF2 expressing cells could control SMC phenotype through EV-mediated transfer of miR-143/145 and in vivo this led to reduced aortic lesion size in apolipoprotein-E (apoE)^−/−^ mice [153]. These data highlight that two different triggers can alter the behaviour of EC-derived EVs, underlining the role of the microenvironment in the biological effects of EVs and why sometimes it is very difficult to decipher their net contribution to the progression of the disease. One possible explanation could be that a different trigger alters the EV cargo and thereby modulates cell-cell communication thus generating a different biological effect.

Another important cell type participating in lesion progression is the monocyte. In many studies it has been reported that monocyte-derived EVs (exosomes and microvesicles) promote vascular inflammation and vascular cell death, often via miRNA transfer or increased cytokine expression (IL-6, IL-1β) and via upregulation of the expression of VCAM-1, ICAM-1 and *E*-selectin in ECs [[154], [155], [156]]. Zhang et al., reported that monocyte-derived EVs can induce EC migration by miR-150 transfer to ECs [157]. Atherosclerotic plaque EVs can transfer ICAM-1 directly to ECs and promote inflammatory cell recruitment suggesting that plaque EVs aggravate lesion progression [158]. Macrophage-derived EVs and in particular their miRNA cargo are of great interest. It has been reported that upon application of the atherogenic stimuli oxLDL, macrophage EVs are enriched for many miRNAs including miR-146a, miR-128, miR-185, miR-365, and miR-503 [159]. Furthermore, miR-146a could accelerate the progression of atherosclerosis by inducing macrophage migration into the vessel wall [159]. Foam cell-derived EVs have been shown to induce SMC migration and extracellular signal-regulated kinases (ERK) pathway activation which could aggravate lesion progression [160].

Platelet-derived EVs have been implicated in phenotypic modulation of immune and vascular cells by interacting with subendothelial elements [161]. Phenotypic modulation of SMC towards a synthetic phenotype is characteristic of the disease. Platelet-derived EVs can induce SMC proliferation and migration by inducing a proinflammatory phenotype in SMCs [162,163]. Proteomic analysis of EVs, revealed that Ras-related protein 1 (Rap1) was overexpressed in patients with metabolic syndrome compared to healthy donors [164]. A larger proportion of Rap1 + EVs originating from platelets, promoted SMC migration and proliferation probably via ERK5 activation in vitro and these effects were abolished after Rap1 inhibition [164]. Elevated levels of Rap1 were detected on circulating EVs from high fat fed apoE^−/−^ mice too [164]. Evidence also suggests that they are responsible for monocyte recruitment to the atheroprone endothelium [165]. Transcriptomic analysis of platelet and platelet-derived EVs during senescence revealed that several miRNAs in platelet-EVs (miR-144-3p, miR-486-5p, miR-142-5p, miR-451a, miR-25-3p, miR-145-5p, and let-7f-5p) could target components of lipid metabolism, the inflammatory response and coagulation [166].

Regarding macrophages, studies have shown that platelet EVs can reduce macrophage reactivity by altering their differentiation into the M2 phenotype [167,168]. Laffont et al., demonstrated that platelet EVs transfer miR-126–3p to macrophages resulting in alteration of their gene expression profile (ATF3, ATP1B1, ATP9A and RAI14 expression), downregulation of inflammatory cytokine production (CCL4, CSF1 and TNF) and an increase in macrophage phagocytic capacity [169]. Importantly, platelet-derived EVs are also reported to have both pro- and anti-inflammatory effects in recipient cells. Although isolation techniques can also alter the biological effect of isolated EVs, the previously discussed studies used differential centrifugation for EV isolation. Therefore, the diverse biological responses may be explained by varied target cells and the different cargo being transferred from platelet-derived EVs.

Intercellular communication between EC and SMC is important in maintaining vascular homeostasis. The X-box binding protein 1 (XBP1) is crucial for the vascular functions of EC and SMC [170]. XBP1 splicing can lead to miR-150 EV-mediated transfer from SMC to EC and activate the VEGF-A/VEGFR/PI3K/Akt pathway that regulates EC migration [170]. Inhibition of miR-150 transfer suppressed the EC-migratory effect [170]. Moreover, miR-155 EV mediated transfer from KLF5-transduced SMCs to ECs resulted in the destruction of tight junctions and endothelial barrier integrity and promoted atherosclerosis and infusion of these EVs into high fat fed apoE^−/−^ mice resulted in larger size plaques, an effect inhibited by transfer of miR-155 [97]. MiR-143 and miR-145 transfer from KLF2-induced endothelial EVs blocked SMC transdifferentiation providing an atheroprotective effect mediated through EC-SMC communication [153]. MiRNAs have attracted a lot of interest as inhibition of EV-mediated miRNA transfer has proved to be an effective therapeutic target in atherosclerosis [97].

### 3.2 EVs and plaque stability

The need to find biomarkers that would enable an assessment of endothelial dysfunction and the possibility of a coronary event is imperative. Endothelial- and platelet-derived EVs are the major circulating particles in blood and studies have shown that they can be used as independent biomarkers for CAD status [[171], [172], [173], [174], [175]]. Activated macrophages undergo necrosis and build up in the plaque's necrotic core. Many signals can promote macrophage necrosis, including EV signalling. A recent study showed that T-cell-derived EVs were cleared by macrophages via phagocytosis and caused macrophage apoptosis [176]. Interestingly, as well as increased apoptosis, EVs also promoted microparticle release by macrophages, possibly generating new apoptotic messages which could then amplify cell death [176]. Studies have shown that T-cell-derived EVs activate the phospholipid-ceramide pathway, production of arachidonic acid and increased levels of proapoptotic ceramides by EVs [177]. However, other studies indicate that platelet, endothelial, dendritic and monocytic-derived EVs encapsulate cell death related proteases: caspase-1 and caspase-3 that can induce macrophage apoptosis [[178], [179], [180], [181]]. SMC death can also increase the plaque necrotic core and therefore plaque size [182]. Monocyte-derived EVs were reported to encapsulate caspase-1 and induce SMC death [181]. Furthermore, T-cell derived EVs promoted cholesterol accumulation into monocytes and macrophages, forming foam cells, unravelling another proatherogenic factor and increasing lipid accumulation in the lipid core [183].

### 3.3 EVs and vascular calcification

Many studies have identified the presence of EVs in calcified plaques and showed their involvement in the process [42,184,185,186]. Endothelial EVs have been reported to have increased calcium and bone morphogenetic protein 2 (BMP-2) levels resulting in promotion of calcification and in BMP-2 inducing an osteogenic phenotype in SMCs [184]. Macrophage-derived EVs are rich in S100A9 and annexin V, molecules that aggravate calcification processes in chronic kidney disease [186]. Chen et al., showed that EVs derived from calcified vascular SMCs could promote changes in neighbouring cells, accelerating calcification via many pathways [187]. A further study defined the EVs secreted from SMCs as exosomes and showed that they mediate vascular calcification [42]. In this study certain atherogenic stimuli such as elevated levels of calcium, tumor necrosis factor-α (TNF-α) and platelet derived growth factor (PDGF) were reported to induce exosome secretion and therefore manipulation of the EV secretory pathway and could comprise a new potential therapeutic target [42]. EV cargo and its role in vascular calcification is of great importance. The role of miRNAs in vascular calcification has been extensively discussed (reviewed in [188]). Lin et al., proposed a similar mechanism where they showed that miR-206 expression in ECs controlled the contractile phenotype of SMCs by inhibiting both exosome secretion from ECs, and miR-26a transfer from ECs to SMCs via EVs, through targeting ARF6 and NCX1 [189]. Collagen in a major component of the fibrous cap and reduced collagen leads to a thin and fragile cap resulting in a vulnerable and unstable plaque [182]. Hutcheson and colleagues, showed in a 3-D in vitro model that EV aggregation increased mineralization and collagen acted as a scaffold for EVs to aggregate and direct the calcification process [48].

Another molecule which has attracted interest in vascular calcification research is Sortilin 1. Sortilin 1 is reported to be localised to calcified human plaques and calcified arteries from patients and in an experimental model with chronic renal disease (CRD) [190]. Furthermore, sortilin 1 ablation rescues vascular calcification with no effect on bone mineralization and moreover ablation of sortilin in mice on a LDL receptor-deficient (Ldlr^−/−^) background reduced vascular calcification by 80% when compared with Ldlr^−/−^ mice expressing sortilin 1 [190]. In vitro experiments in which sortilin was either silenced or overexpressed in human coronary arterial SMCs (hSMCs) confirmed its direct role in vascular SMC calcification [190]. Sortilin 1 was also reported to control transfer of the calcification protein tissue nonspecific alkaline phosphatase (TNAP) into EVs and as a result regulated the impact of EVs on calcification [190]. This study provided a novel mechanism for the production of SMC calcifying EVs and a new therapeutic target to address in vascular calcification.

### 3.4 EVs and vulnerable plaques

Many studies have underlined the role of EVs in atherosclerotic plaque destabilization and thrombus formation. Characterisation of EV content of vulnerable plaques showed that they contained a number of thrombogenic microvesicles mainly originating from leukocytes, macrophages, erythrocytes, lymphocytes and SMC [129,130,191]. Further research highlighted that these EVs were more concentrated in the plaque area and their capacity to generate tissue factor and thrombin was greater in plaque EVs indicating the procoagulant potential of EVs [129]. Fibrous cap rupture exposes such thrombogenic material to circulating platelets activating them and leading to thrombus formation [47]. Finally, circulating monocyte-derived and platelet-derived EVs can enhance thrombus formation [192,193]. Since EVs have phospholipids on their surface it is no surprise they can bind to coagulation factors. However, the thrombogenic effects of EVs in destabilising atherosclerotic plaques are yet to be determined.

Plaque rupture is mainly caused by fibrous cap weakening and studies have suggested that circulating EVs can disturb local inflammation and destabilize atherosclerotic plaque caps [47]. SMC hold a crucial role in forming a thick fibrous cap by secreting collagen, elastin, fibronectin and extracellular matrix [37]. EVs, mainly platelet-derived EVs, have been shown to cause vascular cell death [47,194]. The breakdown of extracellular matrix and destabilization of the plaque has been attributed to MMPs and several studies have now demonstrated that EVs carry different MMPs as their cargo [195]. Macrophage-derived EVs exposed to tobacco were reported to carry MMP14 [134]. EC-derived EVs (both in vitro and in vivo) were shown to carry MMP-2, MMP-10, MMP-9 and MMP-14 [[196], [197], [198]] on their external surface. Characterisation of neutrophil-derived EVs showed that they encapsulate proteolytic enzymes, among them MMP-9 [199]. The cell source of EVs can affect their proteolytic cargo and this may also effect fibrous cap integrity. For example, microvesicles isolated from atherosclerotic lesions expressed the metalloprotease TNF-α converting enzyme (TACE/ADAM-17) [200]. TACE/ADAM17 was previously found on atherosclerotic lesions [200]. ADAM-17 and similar proteases cleave transmembrane molecules such as cytokines. ADAM-17 is responsible for TNF-α secretion by cleaving the precursor of TNF-α (pro-TNF-α) into the soluble cytokine and maintains a balance between anti- and pro-inflammatory molecules [200]. Potential cell sources could be leukocytes or erythrocytes and it was shown that EVs could stimulate in vitro release of TNF-α [200]. EVs isolated from abdominal aortic aneurysm samples were found to be positive for a disintegrin and metalloprotease-10 (ADAM-10) and ADAM17 [201].

## 4 Extracellular vesicles as biomarkers for cardiovascular disease

As previously stated EVs are present in many biological fluids including blood, saliva and urine and in both health and disease [106,202] and have therefore attracted attention as liquid biomarkers. Studies have reported that elevated levels of circulating EVs show a correlation with cardiovascular events in patients with stable CAD [[171], [172], [173], [174]]. In many of these studies, quantification of endothelial-derived EVs was used to predict future coronary events related to endothelial dysfunction [[171], [172], [173]]. Circulating CD31+/Annexin V+ EVs were found to be increased in patients with stable CAD and cardiovascular risk factors (e.g. diabetes) suggesting them as an independent risk factor for cardiovascular outcomes in patients with CAD [174]. Interestingly, circulating endothelial-derived EVs are increased in patients with underlying cardiovascular risk factors [131,132] including smoking [133,134], hyperlipidaemia [135] and hypertension [136,137]. Furthermore, studies suggest that leukocyte-derived EVs could be used as a biomarker to predict subclinical atherosclerosis and the role of leukocyte-EVs in atherosclerosis is reviewed in [203]. Quantitative data showed that patients with acute coronary syndrome (ACS) (and undergoing percutaneous coronary intervention (PCI)) had elevated levels of circulating EVs compared to patients with stable angina [204,205]. Another study showed that leukocyte EVs were found to be elevated in patients with unstable carotid plaques compared to patients with stable carotid stenosis, therefore leukocyte-derived EVs are a potential biomarker to determine plaque vulnerability [206]. Annexin V+ EVs of platelet, endothelial, neutrophil and granulocyte origin were found to be elevated in heterozygous familial hypercholesterolemia (FH) patients with atherosclerotic plaque compared to patients without plaque [207]. Both group of patients were asymptomatic and circulating EVs could predict atherosclerotic burden and cardiovascular events in FH patients [207]. Elevated levels of EV-derived CD14 showed a strong correlation with vascular disease [208]. Lastly, circulating EVs from patients with cardiometabolic syndrome are enriched with Rap1 and could be a surrogate biomarker for early atherosclerosis detection [164]. Taken together these data suggest that EVs could be used as liquid biomarkers to monitor CVD progression.

The active cargo of EVs, mainly miRNAs, is of great research interest with studies correlating levels of EV-miRNA with prediction of CAD events. It was reported that elevated levels of EV miR-126 and miR-199a but not circulating soluble miRNAs were linked with lower adverse cardiovascular events in patients with stable CAD [175]. Another study examined the miRNA levels from atherosclerotic plaques and healthy regions of the artery from patients undergoing heart transplantation and showed decreased levels of EV miR-143-3p and miR-222-3p at lesion sites [209]. Several other EV-derived miRNAs such as miR-133a, miR-143/145, miR-150, miR-155, miR-214, miR-223, and miR-320b have also been reported as biomarkers for atherosclerosis risk prediction (reviewed in [210]). Goetzl et al., reported that the protein cargo of EVs could also be exploited to predict the progression of the disease [211]. In patients with atherosclerotic cerebrovascular disease, EC-derived EVs were enriched with VCAM-1 and PDGF proteins, which was implicated in lesion progression, compared to the healthy control group [211]. These studies highlight the potential use of EVs as biomarkers for monitoring atherosclerosis progression, however further clinical studies in independent cohorts are required to draw definitive conclusions.

## 5 EVs as therapeutic delivery vectors

EVs have attracted a lot of attention as therapeutic delivery vectors in CVD as they may have advantages over current delivery systems which may exhibit limitations such as non specific binding, toxicity and increased clearance and as a result lower therapeutic potential [212]. EVs are normally secreted from cells, thus they present low immunogenicity and their lipid core offers stability [213]. Two types of EV delivery systems can be used: native EVs which are secreted from parental cells or engineered EVs which can be loaded with therapeutic molecules after isolation or can be genetically modified to express cell specific proteins for targeted delivery and to avoid clearance [214,215]. Different mechanisms can be manipulated for drug loading into the EVs such as electroporation or sonication [213]. Certain limitations regarding EV exploitation as therapeutic delivery systems still exist such as drug loading efficacy or cell-specific targeting [216]. Furthermore, EVs may already contain their own endogenous contents which may be difficult to control. Recent studies have investigated the effect of native EVs in cardiocascular disease. Mesenchymal stem cell (MSC)-derived EVs have been used for tissue regeneration such as cardiac regeneration after MI [217] or reperfusion injury [218]. MSC-derived EVs have also gained interest in atherosclerosis treatment. Li et al., reported that treatment of apoE^−/−^ mice under high fat diet with MSC-derived EVs resulted in reduced plaque size and macrophage infiltration to the plaque area [219]. In vitro experiments revealed that EVs promoted macrophage polarization towards the M2 phenotype via the axis miR-let7/IGF2BP1/PTEN and unraveled a potential target for atherosclerosis [219]. Another study, used human bone marrow MSC-derived EVs containing miR-221 to treat apoE^−/−^ mice under high fat diet and reduced plaque size was observed [220]. In vitro and in vivo experiments also revealed that EV mediated miR-126 transfer from ECs to SMCs regulated SMC proliferation and resulted in reduced neointima formation after vascular injury [221]. EVs participate in various steps of atherosclerotic disease progression (as described in section 3). Direct targeting of EV mediated pathological functions or EV loading with therapeutic molecules has been proposed. Inhibition of EV mediated miR-155 transfer from SMCs to ECs reduced plaque size in apoE^−/−^ mice under high fat diet after receiving LNA anti-miR-155 for 4 weeks [97]. Another in vitro study observed that when macrophages and SMC were treated with EC-derived EVs loaded with anti-miR-33a-5p, ATP-binding cassette transporter ABCA1 protein expression was increased and elevated apoAI-mediated cholesterol efflux was observed too [222]. Molecularly engineered EVs for treatment of atherosclerosis have also been investigated [223]. Wu et al., exploited M2 macrophage-derived EVs which were electoporated and loaded with the FDA approved compound, hexyl 5-aminolevulinate hydrochloride (HAL) [223]. Administration of engineered EVs to apoE^−/−^ mice under high fat diet showed reduced lesion area compared to control mice or mice receiving HAL or M2-derived EVs. In vitro experiments revealed that engineered EVs had higher levels of anti-inflammatory cytokines such as IL-10 [223]. EVs as vectors offer an enormous opportunity for the development of new pharmacological therapies and will be an area of further investigation in the future.

## 6 Conclusion

EVs represent a new growing area of research with respect to both understanding and treating CVD. They are naturally occurring secreted particles and thus they are suggested to participate in intercellular signalling. Their biological functions are diverse as they can exert both protective and pathological effects. The lack of a single standard EV isolation method is a great challenge regarding the study of EVs as this could be a factor in the observations that EVs often contribute to diverse and conflicting biological effects. Many studies have reported the presence of EVs in atherosclerotic plaques and characterised the biological functions of certain cell-derived EVs. The cross-talk between ECs and SMCs in atherosclerosis development is still not yet completely understood and requires further research. Finally, EVs have been studied as biomarkers for CVD and as therapeutic vehicles; while some of these findings are promising more work to replicate observations in independent study populations is required. Overall, there remains much to be learned in the recently emerging field of EV signalling in atherosclerosis.

## Funding

E Charla is funded by the 10.13039/501100000274British Heart Foundation (FS/17/63/33485) and the College of Medical, Veterinary and Life Sciences, University of Glasgow. SAN is supported by the British Heart Foundation (Centre of Research Excellence Award RE/18/6/34217).

## Declaration of Competing Interest

The authors declare that they have no known competing financial interests or personal relationships that could have appeared to influence the work reported in this paper.
